# Strong incidence of *Pseudomonas aeruginosa* on bacterial *rrs* and ITS genetic structures of cystic fibrosis sputa

**DOI:** 10.1371/journal.pone.0173022

**Published:** 2017-03-10

**Authors:** Laurence Pages-Monteiro, Romain Marti, Carine Commun, Nolwenn Alliot, Claire Bardel, Helene Meugnier, Michele Perouse-de-Montclos, Philippe Reix, Isabelle Durieu, Stephane Durupt, Francois Vandenesch, Jean Freney, Benoit Cournoyer, Anne Doleans-Jordheim

**Affiliations:** 1 Equipe de Recherche, Bactéries Pathogènes Opportunistes et Environnement, UMR CNRS 5557 Ecologie Microbienne, Université Lyon 1 & VetAgro Sup, Villeurbanne, France; 2 Laboratoire de Bactériologie, Centre de Biologie et Pathologie Est, Hospices Civils de Lyon (HCL), Bron, France; 3 Equipe de recherche, UMR CNRS 5558, INRA, INRIA, Laboratoire de Biométrie et Biologie Evolutive, Université Lyon 1 & ISPB, Lyon, France; 4 CIRI, International Center for Infectiology Research, INSERM U1111, CNRS UMR5308, Université de Lyon, Ecole Normale Supérieure de Lyon, France; 5 Laboratoire de Bactériologie, Centre hospitalier Lyon Sud, Hospices Civils de Lyon, Saint-Genis-Laval, France; 6 Centre de Ressources et de Compétences (CRCM) adulte, Centre Hospitalier Lyon Sud, Hospices Civils de Lyon, Pierre Bénite France, France; 7 Centre de ressources et Compétences (CRCM) enfant, Hôpital Femme Mère Enfant, Hospices Civils de Lyon, Lyon, France; 8 Equipe de recherche Pathogénie des Staphylocoques, INSERM U851, Bron, France; University of North Dakota, UNITED STATES

## Abstract

Cystic fibrosis (CF) lungs harbor a complex community of interacting microbes, including pathogens like *Pseudomonas aeruginosa*. Meta-taxogenomic analysis based on V5-V6 *rrs* PCR products of 52 P. aeruginosa-positive (Pp) and 52 P. aeruginosa-negative (Pn) pooled DNA extracts from CF sputa suggested positive associations between *P*. *aeruginosa* and *Stenotrophomonas* and *Prevotella*, but negative ones with *Haemophilus*, *Neisseria* and *Burkholderia*. Internal Transcribed Spacer analyses (RISA) from individual DNA extracts identified three significant genetic structures within the CF cohorts, and indicated an impact of *P*. *aeruginosa*. RISA clusters Ip and IIIp contained CF sputa with a *P*. *aeruginosa* prevalence above 93%, and of 24.2% in cluster IIp. Clusters Ip and IIIp showed lower RISA genetic diversity and richness than IIp. Highly similar cluster IIp RISA profiles were obtained from two patients harboring isolates of a same *P*. *aeruginosa* clone, suggesting convergent evolution in the structure of their microbiota. CF patients of cluster IIp had received significantly less antibiotics than patients of clusters Ip and IIIp but harbored the most resistant *P*. *aeruginosa* strains. Patients of cluster IIIp were older than those of Ip. The effects of *P*. *aeruginosa* on the RISA structures could not be fully dissociated from the above two confounding factors but several trends in these datasets support the conclusion of a strong incidence of *P*. *aeruginosa* on the genetic structure of CF lung microbiota.

## Introduction

Pulmonary infections represent the main cause of morbidity and mortality among individuals with cystic fibrosis (CF) [1]. These infections are due to well-known pathogens such as *Pseudomonas aeruginosa* and *Staphylococcus aureus* [2, 3]. However, as for patients with respiratory disorders like asthma or chronic obstructive pulmonary disease (COPD), studies based on non-cultural analysis showed the presence of complex lung microbiota in CF patients [4–6]. Prior to causing an infection, CF pathogens must therefore face and invade these microbial communities [7–13].

Several modifications in the structure of CF microbiota can occur, potentially favoring the pulmonary colonization by a pathogen. First, these microbiota evolve over time in terms of genetic diversity, richness and evenness, as shown by the association between patient aging and a decrease of microbial diversity [14, 15]. Second, the genetic structures of microbiota in CF lungs were related to the type of *cftr* mutation [14] and to the pharmacological treatments (antibiotics) [16–18] as well as to the geographic location of the patients [19] and to the exposition to environmental stressors such as pollutants or pets [20, 21]. Third, clinical status and particularly CF exacerbation can induce significant changes in the CF microbiota and lead to and/or result from an evolution from a stable to a disturbed state [22].

Among factors leading to modifications in the structure of CF microbiota, several bacterial properties that favor microbial interactions have been proposed. Resistances towards bacteriocines, pyocines and other antimicrobials, as well as sensing of various products such as homoserine-lactones which can trigger the expression of virulence factors, are frequently suggested. For example, *P*. *aeruginosa* can produce 4-hydroxy-2-heptylquinoline *N*-oxide (HQNO) that inhibits the cytochrome oxidase of *S*. *aureus* [23, 24] and subsequently blocks its growth. Antagonistic or mutualistic networks of interactions will then promote CF lungs invaders such as *P*. *aeruginosa* and thus contribute to the pathogenesis of the infections and their outcome [25–29].

To study CF microbiota and their fluctuations, many non-cultural methods are available. Among them, the next-generation sequencing (NGS) technologies, and more particularly the *rrs* (16S rDNA) meta-taxogenomic analyses, allowed defining CF core microbiota at the genus level and suggested several potential mutualistic associations between bacterial groups or operating taxonomic units (OTU) [30]. However, the regions of the 16S rRNA gene used, PCR biases [31, 32], sequencing errors [33] and high numbers of chimeras [34] can have a great impact on the inferred richness and genetic structures [35]. Furthermore, despite being more and more affordable, NGS remains an expensive and time-consuming option [36]. Genetic fingerprinting approaches represent an attractive alternative to these NGS analyses, and were used successfully on CF lung microbial communities [37, 38]. The RISA technique based on peaks profile resulting on the amplification of the noncoding ITS (Internal Transcribed Spacer) between the *rrs* (16S rDNA) and *rrl* (23S rDNA) sequences, is described as highly reproducible, robust and time-efficient [39]. Changes in the size of the ITS PCR products can resolve the genetic diversity of microbiota at the species level [40, 41] but rarely provide identification of the bacteria. Still, some RISA peaks can be indicative of the presence of certain species like *P*. *aeruginosa* [37]. Van Dorst *et al*. (2014) showed that the RISA results were consistent with data produced from the 454 pyrosequencing analysis in terms of microbial community indices (richness and diversity) [39].

In this study, we investigated the incidence of a major pathogen, *P*. *aeruginosa*, on the bacterial genetic structures of CF lungs. We hypothesized that this bacterium can modulate the CF microbiota, potentially by microbial interactions, leading to strong differentiations between positive and negative *P*. *aeruginosa* CF bacterial communities. This hypothesis was built from inferences made by *in vitro* investigations revealing strong antagonisms between *P*. *aeruginosa* and *S*. *aureus* or *Burkholderia cenocepacia* [42, 43]. However, cooperation could also occur, such as the one reported with *Stenotrophomonas maltophilia* which appeared to favor *P*. *aeruginosa* development [44].

Our hypothesis was first tested by a sampling strategy using NGS analysis of pooled DNA extracts from two significant groups of patients (n = 52 per group) differentiated by the presence or the absence of *P*. *aeruginosa*. Then, the impact of *P*. *aeruginosa* on CF microbiota genetic structures was studied through genetic diversity and richness indices computed from RISA profiles. NGS and RISA were complementary, the NGS giving insights on the bacterial groups found among the investigated CF cohorts and RISA giving access to a deeper resolution of the genetic bacterial community structures on a per sample basis. Finally, the incidence of confounding factors such as antibiotic therapies and clinical characteristics of the patients on the RISA datasets was investigated.

## Experimental procedures

### Ethic statement

Ethic approval for this study was granted by the *Commission Nationale de l’Informatique et des Libertés* (CNIL) under the reference 913442. Samples were those from routine bacteriological analyses of CF patients and did not require any additional obligation. Genetic data of the patients were not collected. Written consents of the patients to use their sputum samples were not requested by the ethical group. Oral consents were obtained from all adult patients, and from parents of all young patients. A note was added to the patient record reminding the oral acceptance.

### Sputa collection

A total of 104 sputa were recovered over 3 months from CF patients of Centre de Biologie et Pathologie Est (CBPE) and Centre de Biologie Sud (CBS) of Hospices Civils de Lyon (France). These samples were divided into two groups (n = 52 each), based on whether or not *P*. *aeruginosa* was detected by the “classical” microbiological investigations, with pairing according to the age of the patients. For each patient, age, sex, clinical exacerbations (mentioned in the clinical record or according to clinical criteria defined by Fusch *et al*. (1994) [45]) and antimicrobial treatments received 3 months prior the collection of a sputum were recorded (S1 Table). A total of 61 strains of *P*. *aeruginosa* were isolated from these sputa, and kept for further analyses. Strains PA14 UCBPP bpoe 2262 and PAO1 (Canada) were used for comparison purposes in some analyses.

### General manipulations

Extractions of total bacterial DNAs from sputa and LB broths were performed with the NucleoSpin Tissue^®^ kit (Macherey-Nagel) according to the manufacturer. Sputa were diluted with Sputasol (Thermo Scientific^®^) before DNA extraction. DNA extracts were stained with the PicoGreen dye^®^ and quantified with a NanoDrop 3300 Fluorospectrometer (Thermo Scientific^®^).

Detection of *P*. *aeruginosa* was carried out by the CBPE and CBS using the *ecfX* (sigma factor involved in extracytoplasmic functions) PCR screening [46]. PCR conditions are described in S2 Table. PCRs were performed on a Mastercycler^®^ (Eppendorf). PCR products were visualized by agarose gel (1.5%) electrophoresis, staining with ethidium bromide, and UV light exposure.

### Meta-taxogenomic analysis of 16S rDNA (*rrs*) PCR products

Sputa DNA extracts were pooled into two groups, one containing 5 ng of each DNA extracted from the 52 sputum samples containing *P*. *aeruginosa*, named Pp, and the other one containing 5 ng of each DNA extracted from 52 sputum samples without *P*. *aeruginosa*, named Pn. The V5-V6 *rrs* regions were PCR amplified and sequenced by a 454 Life Sciences Genome Sequencer FLX instrument (Roche) following Titanium chemistry. The generated sequences were filtered using the MOTHUR version 1.35.1 package in order to remove chimeric sequences, primers, barcodes, and limit the dataset to sequences of a minimum length of 200 bp (average length = 260 bp) [47]. A total of 54,668 and 61,854 V5-V6 *rrs* sequences for the *P*. *aeruginosa* positive (Pp) and the *P*. *aeruginosa* negative (Pn) groups could be recovered, respectively. The SILVA 16S rDNA Bacterial (v119) reference library was used for taxonomic allocation of the OTUs. In order to avoid mistakes due the short reads, cut off was set at 0.01 (99% identity) for OTUs classification.

### Ribosomal RNA Intergenic Spacer Analysis (RISA)

The ITS between *rrs* (16S rDNA) and *rrl* (23S rDNA) were PCR amplified from all individual DNA samples according to Nazaret *et al*. (2009) [37]. PCRs were performed according to S2 Table. ITS length variations were measured by injecting the PCR products into a POP-7 polymer containing 36 cm capillary arrays connected to an ABI Prism 3730XL Genetic Analyser^®^ (Applied Biosystems) [48]. Injection time was of 42 s and run voltage was set at 15 kV. Internal control (MRL LIZ 1000) consisted of a panel of fragments of 50 bp to 1,000 bp emitting fluorescence intensities greater than 150 RFUs (Relative Fluorescence Units). Lengths of all ITS PCR products per sample were estimated using the above standards. All PCR products between 100 and 1000 bp were analyzed using the Peak Scanner Software^®^ (Applied Biosytem). Only ITS PCR products giving fluorescence signals greater than 150 RFUs were considered in the RISA process. The relative amount of each ITS fragment per sample was estimated as the ratio between the fluorescence (peak area) of the fragment of interest and the total fluorescence of all fragments in the profile.

### Pulsed field gel electrophoresis

PFGE of *Spe*I restricted total *P*. *aeruginosa* DNAs were performed according to Lavenir *et al*. (2007) [49]. Pairwise comparisons of PFGE profiles were done using the Dice correlation and clustering analyses with the Unweighted Pair Group Mathematical Average (UPGMA) clustering algorithm of BioNumerics version 6.0 (Applied Maths, Austin, TX, USA). PFGE-*Spe*I profiles were interpreted according to the guidelines proposed by Römling *et al*. (1994) [50] and Tenover *et al*. (1995) [51]. Profiles having less than six band changes were merged into clones or clonal complexes according to Lavenir *et al*. (2007) [46]. PFGE profiles showing six or more band changes were considered as ‘different,’ and, thus, not to have a common recent ancestor that could be inferred by this approach.

### Antimicrobial susceptibility testing

Antimicrobial susceptibility of the clinical isolates was determined by the disk diffusion method on Mueller-Hinton II agar as recommended by the Antibiogram Committee of the French Society for Microbiology (CA-SFM: Comité de l’Antibiogramme de la Société Française de Microbiologie) [52]. The 14 antimicrobial drugs tested (all from Bio-Rad) were *(i)* beta-lactam antibiotics class: ticarcillin (TIC, 75 μg), ticarcillin-clavulanic acid (TCC, 75 / 10 μg), piperacillin (PIP, 75 μg), piperacillin-tazobactam (PPT, 75 / 10 μg) ceftazidime (CAZ, 30 μg), cefepime (FEP, 30 μg), aztreonam (ATM, 30 μg), imipenem (IPM, 10 μg) and meropenem (MEM, 10 μg), *(ii)* aminoglycosid antibiotics class: gentamicin (GMI, 15 μg), amikacin (AKN, 30 μg), tobramycin (TMN, 10 μg), *(iii)* fluoroquinolone antibiotics class: ciprofloxacin (CIP, 5 μg) and *(iv)* other antibiotic class: fosfomycin (FF, 50 μg + 50 μg G6P).

### Statistical analyses

For the meta-taxogenomic dataset, differences in genus distribution among Pp and Pn groups were tested by applying Fisher’s exact tests on an OTUs contingency table. For RISA profile analyses, microbiota were clustered using the clustering algorithm described by Ishii *et al*. (2009) [53]. Dendrogram was generated using pvclust package from R software (version 3.1.3) using euclidean correlation and ward method [54]. The RISA datasets were also used to compute diversity indices like Shannon-Wiener (*H*) and Simpson’s discriminatory (*D*_*1*_) indices [55].

Group comparisons were performed with the R software using standard statistical tests. To decide to use parametric or non-parametric tests, a Shapiro-Wilk test was used. Relative proportions of the groupings were compared using Chi2 or Fisher’s exact tests with Bonferroni correction for multiple testing, as necessary. For the comparative analysis of the number of reads per genus between the groups of patients harboring or nor *P*. *aeruginosa*, OTU defined at 99% identity were considered. This allowed performing a non-parametricWilcoxon Mann-Whitney signed-rank test, for example, on the number of reads allocated to *Haemophilus* but divided into OTU-a, OTU-b, OTU-c, …, OTU-x, per group of patients (with or without *P*. *aeruginosa*). The Wilcoxon Mann-Whitney rank test compares two related samples to assess whether their mean ranks differ when a normal distribution cannot be assumed. Reads per OTU were thus considered as replicates representative of the trends observed among the genus. This test was performed independently for all genera. Comparisons of means (including mean diversity indices) were also carried out using univariate Mann Whitney tests but also Student t-tests or one-way ANOVA followed by Tukey's honest significant difference tests, and Kruskal-Wallis tests followed by Steel-Dwass post-hoc tests. These analyses were performed with R software [54] and FactoMineR packages [56]. Spatial representations using confidence ellipses illustrated differences between clusters at p<0.05. The significance of factors such as age, antibiotic therapies, and presence of *P*. *aeruginosa* on the structuration of RISA datasets was evaluated by a redundancy (RDA) analysis.

## Results

### V5-V6 *rrs* meta-taxogenomic analysis of CF sputa

CF sputa were recovered from 104 patients. These sputa were divided into two groups, respectively, named the Pp group for *Pseudomonas* positive sputa and the Pn group for the *Pseudomonas* negative ones. A *rrs* meta-taxogenomic analysis was performed on the two groups using pooled DNA extracts. Working on pooled DNA extracts had the advantage of giving a general picture of *rrs* genetic diversity, and reducing the impact of confounding factors on the datasets. A total of 116522 V5-V6 *rrs* reads was generated, and 89% of these passed the various quality filters and chimera detection processes used in this study. Number of V5-V6 *rrs* reads per group was normalized at 45863 reads. These reads were classified into 924 OTUs (using a threshold of identity set at 99%). Only a few OTUs could not be assigned to a genus (0.03% for the Pp group and 0.06% for the Pn group), and were then considered as part of a group named “unclassified”. Not considering the “unclassified”, 47.4% of reads from Pp group, were affiliated to *P*. *aeruginosa*-like OTUs and 0.04% among the Pn group. To test the incidence of the over-representation of *P*. *aeruginosa* in the Pp datasets, all *P*. *aeruginosa*-like OTUs were removed. This reduced the number of reads to a total of 24130 in the Pp group. The number of reads in the Pn group was normalized accordingly. The removal of *P*. *aeruginosa*-like OTUs changed the proportions of OTUs per phyla and genera for the Pp sputa. Then, 754 OTU (cutoff of 1%) were found among the V5-V6 *rrs* database. Venn diagrams (not shown) revealed that among these OTUs, Pp and Pn groups shared 162 OTUs, but 321 and 271 were specific of each one.

**Fig 1:**
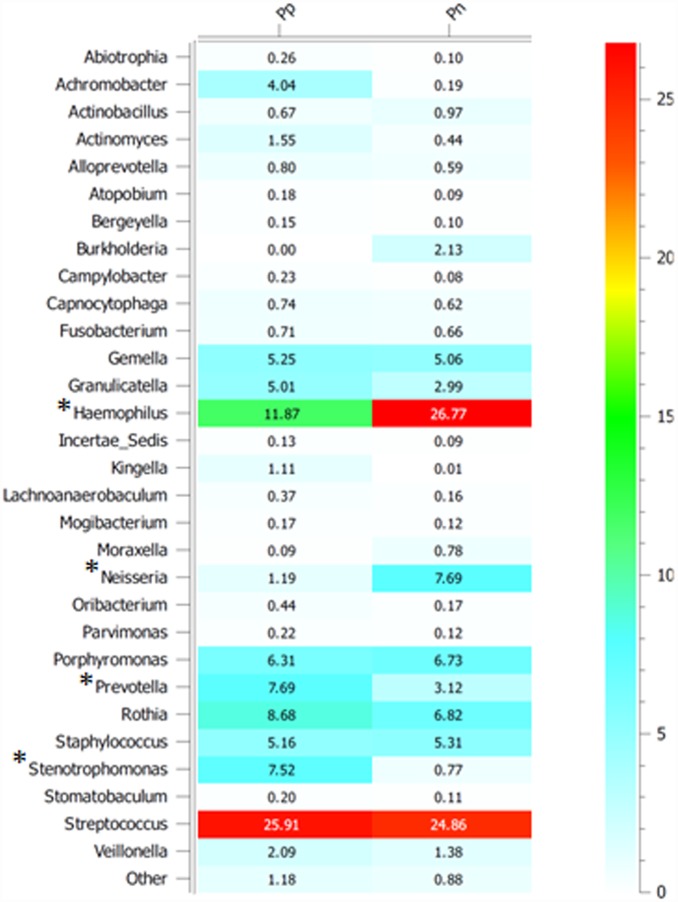
Proportion of V5-V6 *rrs* sequences per genera over the full dataset but without considering reads classified in the *Pseudomonas*. Pp: sputa DNA extracts from CF patients colonized by *P*. *aeruginosa*. Pn: sputa DNA extracts from CF patients not colonized by *P*. *aeruginosa*. Only genera representing more than 0.1% of the full library were considered. * Significant differences (p<0.05) between the Pp and Pn groups were detected by univariate Wilcoxon Mann-Whitney statistical tests while considering reads of each OTU identified per genera (with n OTU > 5 per genera); see materials and methods for the computing, and S3 Table for the number of reads per genera and variance analyses of read numbers per OTU among a genus.

The *rrs* genetic structures between the Pp and Pn sputa (without the *Pseudomonas* reads) were further analysed by investigating the taxonomic allocations of the OTUs. Representations of the Pp and Pn microbiota are shown in S1 Fig. These inferred microbiota were built from taxa recovered at >3% in the datasets. They showed similar proportions in terms of phyla compositions except for the proteobacteria because of the high number of *Haemophilus rrs* sequences in the Pn dataset. A heat map showing the differences in the proportions of OTU per genera between Pn and Pp was built (Fig 1). This heat map was restricted to genera representing more than 0.1% of the normalized OTUs dataset. Statistical analyses of the differences in the number of reads per OTU in a genus between Pn and Pp sputa were computed by univariate Mann Whitney tests (S3 Table; Fig 1). Between n = 5 to 106 OTUs per genus could be used in these analyses. *Prevotella* and *Stenotrophomonas* OTU showed significant differences in their repartition in the Pp and Pn sputa, revealing a positive association with *P*. *aeruginosa*. *Achromobacter* OTUs showed a similar distribution bias that could not be validated by a statistical test because of a single OTU being involved in this segregation. The Pn sputa showed significantly greater numbers of OTUs for the *Haemophilus* and *Neisseria* genera. Other genera, such as *Streptococcus* and *Staphylococcus* did not reveal significant differences in the distribution of their respective OTUs.

### RISA of individual CF sputum DNA extracts and clustering

**Fig 2:**
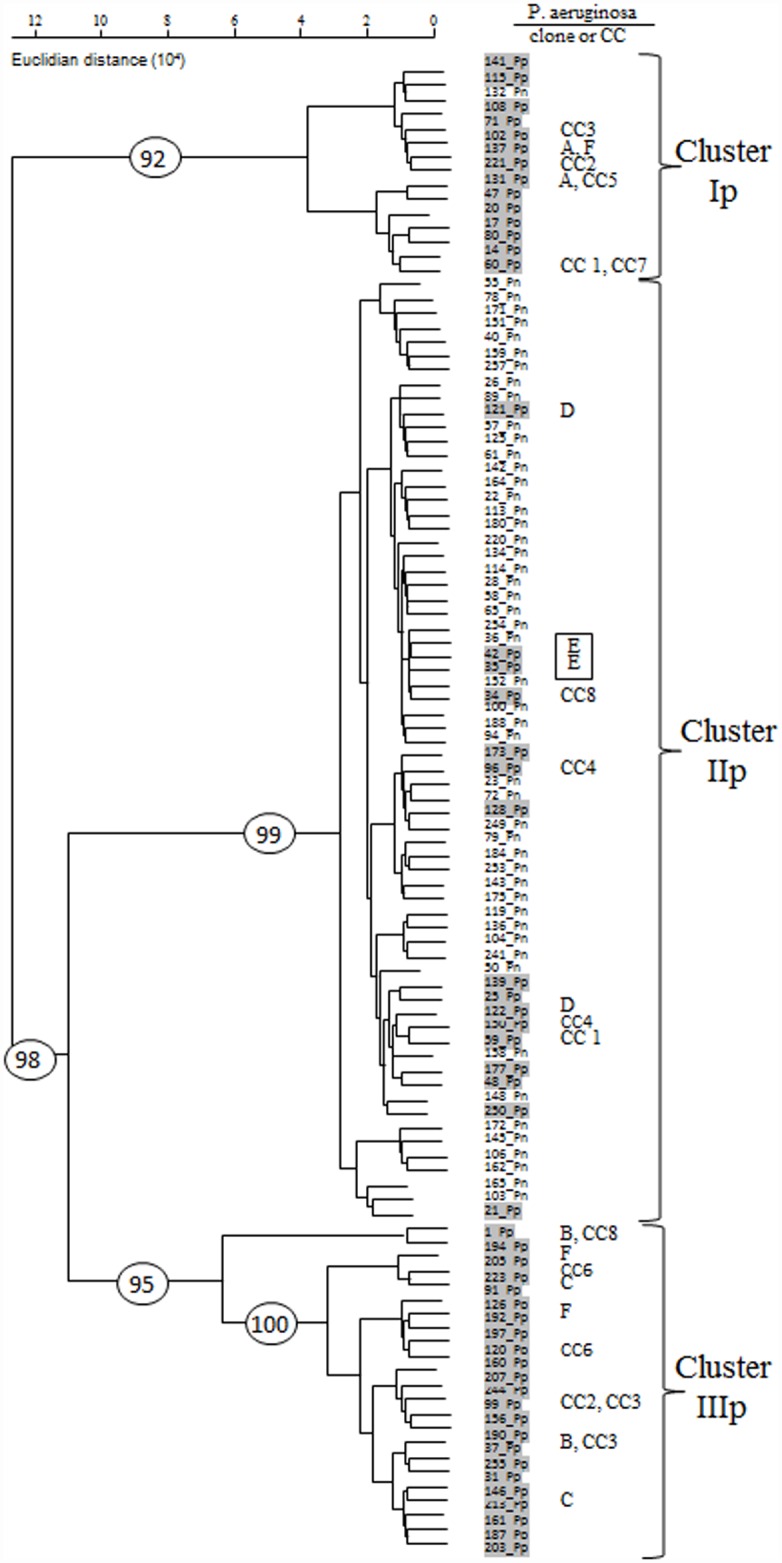
Hierarchical clustering of RISA profiles obtained from 104 independent CF sputa DNA extracts. Pp (highlighted in pale grey): sputa DNA extracts from CF patients colonized by *P*. *aeruginosa*. Pn: sputa DNA extracts from CF patients not colonized by *P*. *aeruginosa*. Three main clusters of RISA profiles were detected. These were supported by significant bootstrap values shown in ovale. PFGE *Spe*I *P*. *aeruginosa* clonal lineages recovered among several CF sputa are indicated. CC: clonal complexes.

All individual 104 DNA extracts from CF sputa were analyzed by RISA (Fig 2). All RISA profiles obtained from the 52 Pp sputa harbored the *P*. *aeruginosa* ITS peak with a length of 560.15 pb (SD = 0.55). Comparisons of mean numbers of RISA bands per sample suggested the Pp profiles to have a lower richness than those of the Pn sputa (mean of 6.6 versus 10.8 RISA bands / sample Student t-test p = 1.6 10^−04^). Indeed, Pp and Pn microbiota presented respectively a total of 94 and 154 RISA bands. The Pp microbiota were composed of 64.8% of shared RISA bands that one could consider the “core” species of this group of patients, and the Pn microbiota were found to share RISA bands at about 39.6% (Chi2 test, p = 8.4 10^−05^).

A dendrogram was computed to compare the 104 RISA profiles from the Pp and Pn sputa (Fig 2). Three clusters (Ip, IIp and IIIp) were observed with bootstrap values higher than 90%. A redundancy analysis (RDA) was also performed on the RISA dataset, and similar groupings were observed on the two main explanatory axes illustrating 92% of the inertia (see section on confounding factors for further details). Clusters Ip and IIIp showed a significantly higher proportion of Pp sputa than cluster IIp (Chi2, p = 5.8 10^−12^) (Fig 2). The *P*. *aeruginosa* ITS peak was found in 14 out of 15 RISA profiles of cluster Ip (93%, matching the outlier indicated above), in 16 out of 66 profiles of cluster IIp (24.2%) and in all of the 23 profiles of cluster IIIp (100%). Among the Pp sputa, the proportions of the *P*. *aeruginosa* ITS peak (inferred from the surface areas) gave a higher mean value over the surface area of all peaks among the Ip (73.8% (SD = 0.22)) and IIIp sputa (76.6% (SD = 0.23)) than those of the IIp ones (22.8%, SD = 0.26) (% *P*. *aeruginosa* Ip vs IIp, p = 1.2 10^−05^; % *P*. *aeruginosa* IIIp vs IIp, p = 1.3 10^−05^). The RISA profiles from clusters Ip and IIIp harbored higher proportions of *P*. *aeruginosa* ITS peak surface (p = 0.927).

Cluster IIp was significantly richer than the other two with a mean of 10.43 RISA bands per sample, whereas clusters Ip and IIIp had means of 6.53 and 5.43 RISA bands per sample respectively (Steel Dwass tests, IIp vs IIIp, p = 1.1 10^−04^; IIp vs Ip, p = 2.49 10^−02^). No significant difference was found in the ITS richness of clusters Ip and IIIp (p = 7.9 10−^01^). In the same way, the cluster IIp was the most diverse (Shannon index, Steel Dwass tests IIp vs Ip, p = 4.05 10^−02^; IIp vs IIIp, p = 1.1 10^−04^; Simpson D index, Steel Dwass tests IIp vs Ip, p = 4.5 10^−02^; IIp vs IIIp, p = 1.14 10^−04^). No significant difference between the diversity indices of clusters Ip and IIIp was observed. (Shannon index, Steel Dwass tests Ip vs IIIp, p = 4.10 10^−01^; Simpson D index, Steel Dwass tests Ip vs IIIp, p = 8.8 10^−02^) (S2 Fig).

### Incidence of *P*. *aeruginosa* genotypes on RISA profiles

With PFGE analysis, 17 *P*. *aeruginosa* strains were recovered from the 11 sputa of patients belonging to RISA cluster Ip, 17 strains from the 11 sputa of RISA cluster IIp and 26 strains from the 18 sputa of RISA cluster IIIp. Eight clonal complexes (CC1 to CC8) and 6 clones (clone A to F) were identified; each from at least 2 sputa from 2 distinct patients (S3 Fig). Three of the 6 clones (C, E and F) belonged to larger clonal complexes: CC2, CC4, CC5 respectively. The strains belonging to CC1, CC2, CC3, CC5 and CC8 were recovered from two RISA clusters (Fig 2). However, clones were most often restricted to a single cluster of RISA profiles (exception for clone G). Clone E was recovered from two patients which shared closely related RISA profiles (Fig 2).

### Analysis of confounding factors

The effects of factors, other than the presence of *P*. *aeruginosa*, that might have contributed to the observed RISA profiles were investigated. The tested confounding factors were antibiotic treatments, antibiotic susceptibilities and those linked to the patient (sex, age, clinical status).

#### Antibiotic treatments

A significant difference between the mean number of antibiotics received (per patient) per RISA cluster was detected (ANOVA, p = 2.2 10^−05^). Patients of cluster IIp (dominated by Pn sputa) received significantly less antibiotics than patients of clusters Ip and IIIp (Tukey HSD tests, IIIp versus Ip, p = 0.05; IIIp versus IIp, p < 1 10^−04^). However, only patients of cluster IIp received amoxicillin-clavulanic acid (S1 Table). Higher proportions of cluster IIIp patients were treated with colistin aerosol (Chi2 test, p = 3.07 10^−02^), tobramycin IV (Chi2 test, p = 7.94 10^−02^) and piperacillin-tazobactam IV (Chi2 test, p = 3.8 10^−02^). The proportions of patients from cluster IIp treated with at least one anti-*Pseudomonas* antibiotic (aerosol: colistin and tobramycin; oral: ciprofloxacin, gentamicin; intraveneous: ceftazidime, tobramycin, piperacillin-tazobactam and meropenem) were less important (50%) than the one of patients from cluster Ip (93%) and cluster IIIp (87%) (Chi2, p = 2.54 10^−04^). Moreover, higher proportions of patients of cluster IIIp (34.8%) and cluster Ip (26.6%) (Chi2, p = 0.59) received intravenous anti-*P*. *aeruginosa* antibiotics than patients in cluster IIp (6.5%) (Chi2 test, p = 1.9 10^−03^). A negative correlation was found between the mean numbers of ITS peaks per sputa and the mean numbers of antibiotics received by patients (Pearson test, p = 2.4 10^−03^, corr = −0.295). A constrained RDA ordination of the RISA datasets, using significant categorical variables (explaining about 20% of the inertia), was performed. The Akaike information criterion showed only age of patients at time of analysis, the presence of *P*. *aeruginosa* or not in the profile, and of nebulized antibiotics to have a significant contribution on the RDA ordinations. This analysis did not show a significant impact of antibiotics on the RISA profile ordinations.

#### *P. aeruginosa* antibiotic susceptibilities

**Fig 3:**
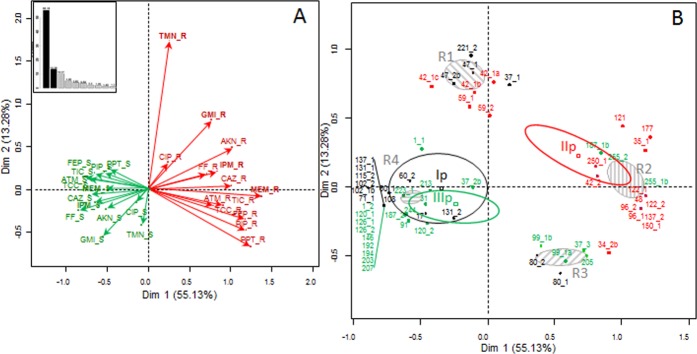
Multiple correspondence analysis of antibiotic susceptibilities among (A) *Pseudomonas aeruginosa* isolates recovered from the sputa, and (B) their matching RISA clusters (Ip, IIp and IIIp). Resistances are in red (antibiotic_R) and susceptibilities in green (antibiotic_S). Percentage of inertia are shown in the corner (A). Confidential ellipses on the panel B represent the statistical distinction of the cluster Ip (black), IIp (red) and IIIp (green) and the 4 resistance patterns (R1, R2 R3 and R4 in grey) observed among the *P*. *aeruginosa* strains (with 5% threshold). Antibiotics: (i) beta-lactams: TIC, ticarcillin; TCC, ticarcillin-clavulanic acid; PIP, piperacillin; PPT, piperacillin-tazobactam; CAZ, ceftazidime; FEP, cefepime; ATM, aztreonam; IPM, imipenem and MEM, meropenem, (ii) aminoglycosids: GMI, gentamicin; AKN, amikacin, TMN, tobramycin (iii) fluoroquinolones: CIP, ciprofloxacin and (iv) FF, fosfomycin.

The antibiotic resistance profiles of 60 strains of *P*. *aeruginosa* were performed and represented using a multiple correspondence analysis (MCA) (Fig 3). The qualitative variables were the fourteen antibiotic susceptibilities tested (resistant (R) and susceptible (S)). The spatial analysis represented 68.5% of the total variability of the antibiotic resistance of the strains (dimension 1: 55.15%, dimension 2: 13.39%) (Fig 3). The projection of the variables highlighted along axis 1 a gradient of antibiotic resistances with antibiotic susceptibilities on the left. The individual representation showed statistical distinctions of antibiotic resistances between the strains following their RISA-clusters. Three confidential ellipses drawn for each RISA-cluster described a similar and lower potential of antibiotic resistances for strains belonging to RISA clusters Ip and IIIp. RISA cluster IIp displayed higher antibiotic resistances. Nevertheless, the individual projection showed a structuration of the strains in four groups (Fig 3). Strains of cluster IIp were divided into 2 groups where the most impacting antibiotic was tobramycin: group R1 (TMN-R, GMI-S, MEM-S and beta-lactams S) and group R2 (TMN-S, GMI-R, MEM-R and beta-lactams R). Strains of cluster Ip harbored two resistance groups, R4 and R1, according to resistance abilities toward TMN with R4 being the most represented one (TMN-S, GMI-S, MEM-S and beta-lactams S). In another way, strains of cluster IIIp were divided into two resistant groups according to their resistance abilities towards beta-lactams. Group R4 was the most represented over cluster IIIp and displayed high susceptibilies to beta-lactams. On the contrary, group R3 exhibited high resistances to beta-lactams (TMN-S, GMI-S, MEM-S and beta-lactams R). However, Chi2 analyses showed cluster IIp to have the highest numbers of MDR-R2 group strains (resistant to at least 5 antibiotics among ceftazidime, cefepime, piperacillin-tazobactam, imipenem, gentamicin, amikacin, ciprofloxacin) (75%) as compared to cluster Ip (11%; Chi2, p < 0.001) and to cluster IIIp (25%; Chi2, p < 0.001).

#### Other factors

**Fig 4:**
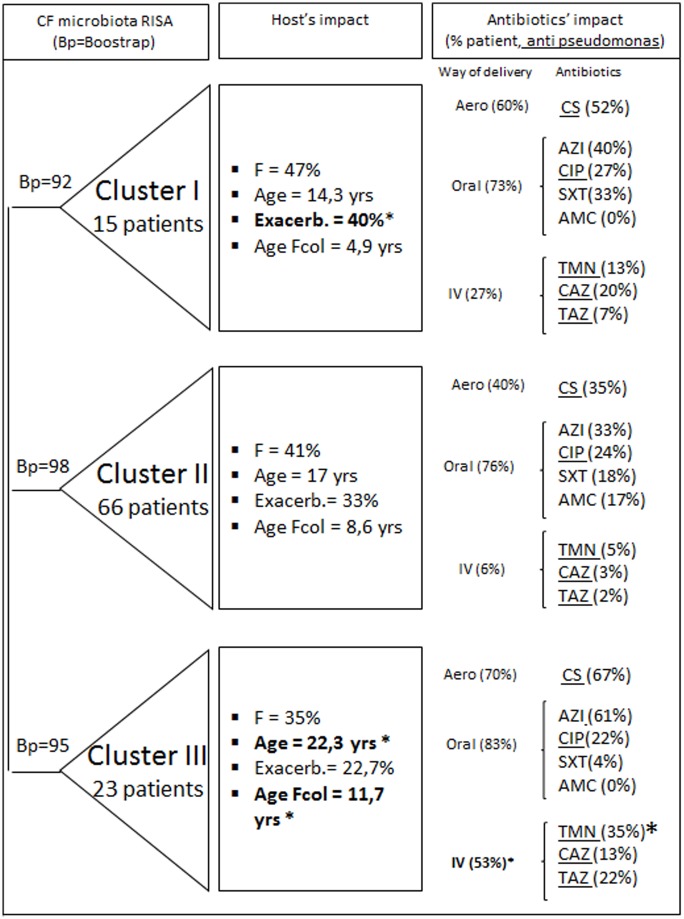
Confounding factors associated with RISA clusters Ip, IIp and IIIp. A significant difference between the proportion or median of age of patients was indicated with * (Fischer exact test, Kruskall Wallis test, p<0,05). F = Female, Fcol = age of the first onset of *P*. *aeruginosa* colonization; Exacerb. = clinical exacerbation. Aero = aerosol, IV = intraveneous. AMC, amoxicillin-clavulanic acid; CAZ, ceftazidime; TAZ, Tazocillin; CIP ciprofloxacin; CS, colistin; TMN, tobramycin; AZI, azithromycin; SXT, sulfamethoxazole-trimethoprim.

The RISA clusters could not be differentiated according to the sex status of the CF patient (non significative Chi2 test). However, patients of cluster IIIp were older than those of clusters Ip and IIp (Steel Dwass tests, IIIp vs IIp, p = 1.97 10^−02^; IIIp vs Ip, p = 1.1 10^−02^). Patients of cluster Ip were colonized earlier than those of cluster IIIp (Steel Dwass tests Ip vs IIIp; p = 4.1 10^−02^) but at about the same age as those of cluster IIp (8.6 years in average; Ip vs IIp, non significative Steel Dwass test). According to the clinical status of the patients, and more particularly the exacerbation symptomatology as defined by Fuchs *et al*. (1994) [45], patients in cluster Ip had more exacerbations (41%) than patients of clusters IIp (22%) and IIIp (15%) (Chi2, p = 0.022) (Fig 4). RDA constrained ordination of RISA profiles using age of patients at time of sampling (and other categorical explanatory variables, see section above) was performed. This analysis showed age to be related to the segregation of clusters Ip (mainly children) and IIIp (mainly adults) but not IIp (data not shown). This is in line with the above descriptions concerning the effect of age on the differentiation of RISA profiles into three clusters.

## Discussion

It is now well-established that human microbiota can interfere with the development of infections, especially in pulmonary diseases such as those observed in cystic fibrosis [4, 57, 58]. The organization of CF microbiota (containing between 20–1000 bacterial taxa) can thus be used as a prognosticator of lung colonization by pathogen and then disease progression [13].

The V5-V6 *rrs* meta-taxogenomic analyses performed in this work allowed identifying key bacterial associations among the CF Lyon cohorts. Some genera were found preferentially associated with *P*. *aeruginosa* such as *Stenotrophomonas*, *Prevotella* and potentially *Achromobacter*. Some bacterial taxa were found absent or at a much lower number among CF sputa containing *P*. *aeruginosa* such as *Haemophilus*, *Neisseria* and *Burkholderia*. The *Stenotrophomonas-Pseudomonas* association likely took place because of synergies or compatibilities between these two species towards the occupation of a same biotope [44]. However, this association might be related to other factors. The presence of *P*. *aeruginosa* leading to a drastic antibiotic treatments, could have favored *S*. *maltophilia* which is characterized by high level of antimicrobial resistances [59] including anti-*P*. *aeruginosa* antibiotics. Even if the incidence of *S*. *maltophilia* on the patients’ status remains unclear [60], these results highlighted an association and/or succession between two non-fermenting CF pathogens in CF lungs that should be followed by clinicians.

In this study, a negative correlation between the presence of *Pseudomonas* and *Haemophilus* was observed, and confirmed previous reports [61]. This suggests that these two genera are antagonistic. *Haemophilus* could limit the pulmonary invasion/infection by *Pseudomonas*. On one hand, a loss of *Haemophilus* could thus be considered a key point in the bacteriological history of the CF lung, giving *Pseudomonas* a greater probably of getting established. In this case, the loss of *Haemophilus* should be tracked and considered as a risk factor and/or a biomaker of a future *Pseudomonas* infection. On the other hand, *Pseudomonas*, colonizing the lungs, could be inhibiting and eradicating *Haemophilus*. A loss of *Haemophilus* could have occurred at the same time as the *Pseudomonas* got successful in their colonization of the CF lungs. Even though both hypotheses are supported by the changes observed over time (*Haemophilus* being found in young patients and *Pseudomonas* in older ones [61]), additional experiments will now be required to validate these trends. Competition/antagonism assays and temporal analyses (before and after *P*. *aeruginosa* arrival) would be also required. It is to be noted that *Neisseria* OTUs were found seven times more abundant in the non-*Pseudomonas* group which was also containing higher *Haemophilus* OTUs, confirming the observations made by Roger *et al*. (2015). Interestingly, *Burkholderia* was never associated with *P*. *aeruginosa* in the CF sputa that were analyzed. This suggested possible incompatibility that would also need to be further investigated.

RISA profiles showed a division of the CF microbiota matching the presence or not of *P*. *aeruginosa*. Clusters Ip and IIIp are dominated by sputa containing this species but not cluster IIp (*P*. *aeruginosa* prevalence of 24.2%). This segregation is in line with the antagonisms inferred from the *rrs* meta-taxogenomic dataset. *P*. *aeruginosa* could produce substances that inhibit some CF bacteria [24–25] and promote others. In fact, this species can greatly affect the nature of the metabolites and substrates that can be found among CF lungs. These compounds could be the key factors explaining the observed CF lung microbiota. They would need to be investigated further in order to better understand their mode of action, and to elaborate new strategies to slow down a CF lung invasion by *P*. *aeruginosa*.

RISA profiles suggested *P*. *aeruginosa* to be associated with a loss of richness in the CF microbiota. Similar trends were previously described by Cox *et al*. (2010) [62] and Klepac Ceraj *et al*. (2010) [14], but not by Price *et al*. (2015) [63]. *P*. *aeruginosa* is a bacterium frequently identified in teenagers and adult CF patients [64]. Microbiota in older patients were described as less diverse than those in young patients [54] and this was associated in most cases with the presence of a high proportion of *P*. *aeruginosa* in sputa. *P*. *aeruginosa* could be responsible for this richness and diversity decrease as the implementation of this pathogen could likely eradicate other micro-organisms. However, it could also be the other way round as a preceding decrease of richness and diversity of lung microbiota could favor a *P*. *aeruginosa* lung colonization. Again, a longitudinal study would be necessary to validate one of these two hypotheses.

The RISA profiles allowed dividing the CF microbiota into three clusters (Ip, IIp, and IIIp) with two (Ip and IIIp) harboring high proportions of *P*. *aeruginosa*-positive samples. Their microbiota showed richness and diversity indices lower than those of cluster IIp. Even though lung microbiota were previously considered highly different between CF patients [8], the presence of three clusters indicated that similarities could be inferred. This was in line with the *rrs* meta-taxogenomic analyses which showed 162 OTUs (over a total of around 300) to be conserved among all the CF sputa analyzed. Furthermore, it is noteworthy that a *P*. *aeruginosa* clone, named E, was found associated with the emergence of two very close RISA genetic organizations. Isolates of this clone had distinct profiles of antibiotic resistance abilities. Their matching patient had not the same age and were exposed to different antibiotics. These observations thus indicate that it could be the nature of this clone which led to the observed microbiota. More investigations on the incidence of *P*. *aeruginosa* clones on the CF microbiota structures will be required to verify this hypothesis but such clinical contexts are quite hard to identify. Still, the presence of specific clones or clonal complexes could not explain alone the RISA clusters that were inferred. In fact, the presence of the *Pseudomonas* species (whatever the clone) clearly appeared to have played part in this differentiation of the CF lung communities into RISA clusters and more particularly between cluster IIp and the other clusters. However, some confounding factors such as age of the patients, clinical status and antibiotic treatments could have contributed, at least in part, to the segregation of the RISA profiles in the above three groups.

One of the risk factors for pulmonary exacerbations reported in literature include infections with *P*. *aeruginosa* [65, 66], even if the presence of *P*. *aeruginosa* (mucoid or not) was not always predictive of lung function decline (major factor describing exacerbation) [8, 67]. However, as suggested by the distinct clinical status of patients from cluster Ip and from cluster IIIp, the presence of *Pseudomonas* in CF lungs could not explain alone these exacerbations. Sibley *et al*. (2008) demonstrated the existence of microbiota that can increase the virulence of *Pseudomonas*. The microbiota of cluster Ip could be part of them [28]. This would explain the higher number of exacerbations associated with this cluster.

A major confounding factor in these investigations was the antibiotic therapy. As confirmed with our study, these antibiotic treatments can decrease the richness and diversity of the CF microbiota and disturb their structures [18, 68]. These observations must be taken into account when investigating indices as predicating factors of the status of a patient. In our study, patients from cluster IIIp received more antibiotic treatments and, in particular, tobramycin and colistin aerosol (both targeting *P*. *aeruginosa*) than patients from cluster Ip, leading to the hypothesis that the nature of the antibiotic treatments could have divided the *Pseudomonas* RISA profiles into two clusters (Ip and IIIp). Surprisingly, strains from cluster IIp, which presented higher proportions of resistances towards antibiotics as well as the highest proportion of MDR *P*. *aeruginosa* [69], were from patients that had received the lowest number of anti-*P*. *aeruginosa* treatments. Still, these comparisons of antibiotic treatments were based on antibiotics used during the immediate previous trimester and older therapies could have contributed to the observed these resistances. Gibson *et al*. (2003) associated the emergence of *P*. *aeruginosa* strains resistant to aminoglycosids to long-term nebulized tobramycin treatments [70]. This was not observed in our study where the highest proportions of tobramycin resistance were found for cluster IIp patients that were the least treated with tobramycin aerosols. Nevertheless, tobramycin resistance remained a discriminative factor which divided *P*. *aeruginosa* strains of cluster II into two resistance groups, strains of cluster IIp resistant to tobramycin being less resistant to other beta-lactams. These results showed antibiotics treatments to have contributed at the modifications of the investigated microbiota but not to be the only significant factor.

It is well-known that the first lung colonization by *P*. *aeruginosa* is a turning point in the life of a CF patient [71] with about 80% becoming chronically colonized by *P*. *aeruginosa*. However, patients who get *P*. *aeruginosa* in their lung later in their life have a better life prognostic [72]. In our study, the age of the first onset of *P*. *aeruginosa* colonization was higher for patients in the cluster IIIp than those of cluster Ip. This difference could be due to several factors. Among these, patients of cluster IIIp could have harbored microbiota or some taxa bringing some sort of protection against *P*. *aeruginosa*. As demonstrated by the meta-taxogenomic analysis, these could include various strains of *Haemophilus* and *Neisseria*.

## Conclusions

The structure of a CF microbiota is the product of complex web of interactions. Their organization could likely be predicted but their key structuring factors need to be identified. Among these, the status of the patients, their age and their antibiotic treatments could represent major forces. Here, *P*. *aeruginosa* was also found to affect these microbiota. The meta-taxogenomic analysis suggested preferential associations between *P*. *aeruginosa* and some taxa like *Stenotrophomonas* and *Prevotella* and antagonistic ones with *Neisseria*, *Haemophilus* and *Burkholderia*. Biases in these bacterial associations were further supported by the RISA datasets. These RISA datasets obtained on a per patient basis allowed differentiating some confounding factors and the effect of *P*. *aeruginosa* on the inferred genetic structures. Longitudinal studies will now need to be performed to further clarify these associations and move towards an implementation of these typing bacterial community approaches in the evaluation of the clinical status of CF patients.

## Supporting information

S1 Table Percentage of patients of clusters Ip, IIp and IIIp which received the listed antibiotics. (TIF)

S2 Table PCR Primer sequences and amplification programs used in this study. (TIF)

S3 Table Median and interquartile values of read numbers per genus and between groups of CF patients with *Pseudomonas* (Pp) or without *Pseudomonas* (Pn); only genera with 5 OTUs or more were considered. (TIF)

S1 Fig Core microbiota without *Pseudomonas* OTU as inferred from V5-V6 *rrs* sequences representing more than 3% of the dataset. A) sputa DNA extracts from CF patients colonized by *P*. *aeruginosa*. B) sputa DNA extracts from CF patients not colonized by *P*. *aeruginosa*.
(TIF)

S2 Fig Comparison of richness and diversity indices of 104 CF microbiota from the three clusters obtained by RISA analyses. (A) Median of peaks per microbiota according to their cluster repartition. (B) Median of Simpson indices representative of the diversity of microbiota according to their cluster repartition. (C) Median of Shannon index representative of the diversity of microbiota according to their cluster repartition. * significant Kruskall Wallis and Steel Dwass multiple comparison tests (p<0.05*).
(TIF)

S3 Fig Dendrogram of *P*. *aeruginosa* PFGE-*Spe*I profiles. 60 clinical CF strains of *P*. *aeruginosa* and two reference strains PAO1 and PA14 were analysed. This dendrogram was realized according to the Dice coefficient and UPGMA correlation on Bionumercis^®^ software with 2% of tolerance. A cutoff set at less than seven band changes between pairs of profiles (Romling *et al*.,1994) was introduced in the analysis of the PFGE data set and used to identify reliable PFGE *Spe*I groupings. Strains with the same PFGE profiles were named A, B, C, D and E and F. The clonal complexes (<7 differences) were named CC1 to CC8. Strain codes in yellow, grey and red belonged respectively to RISA cluster Ip, IIp and IIIp.
(TIF)
