# Supplementary figures and images for: Strong incidence of Pseudomonas aeruginosa on bacterial rrs and ITS genetic structures of cystic fibrosis sputa
Source: PLoS One. 2017 Mar 10;12(3):e0173022. doi: 10.1371/journal.pone.0173022 (PMC5345789; doi:10.1371/journal.pone.0173022)

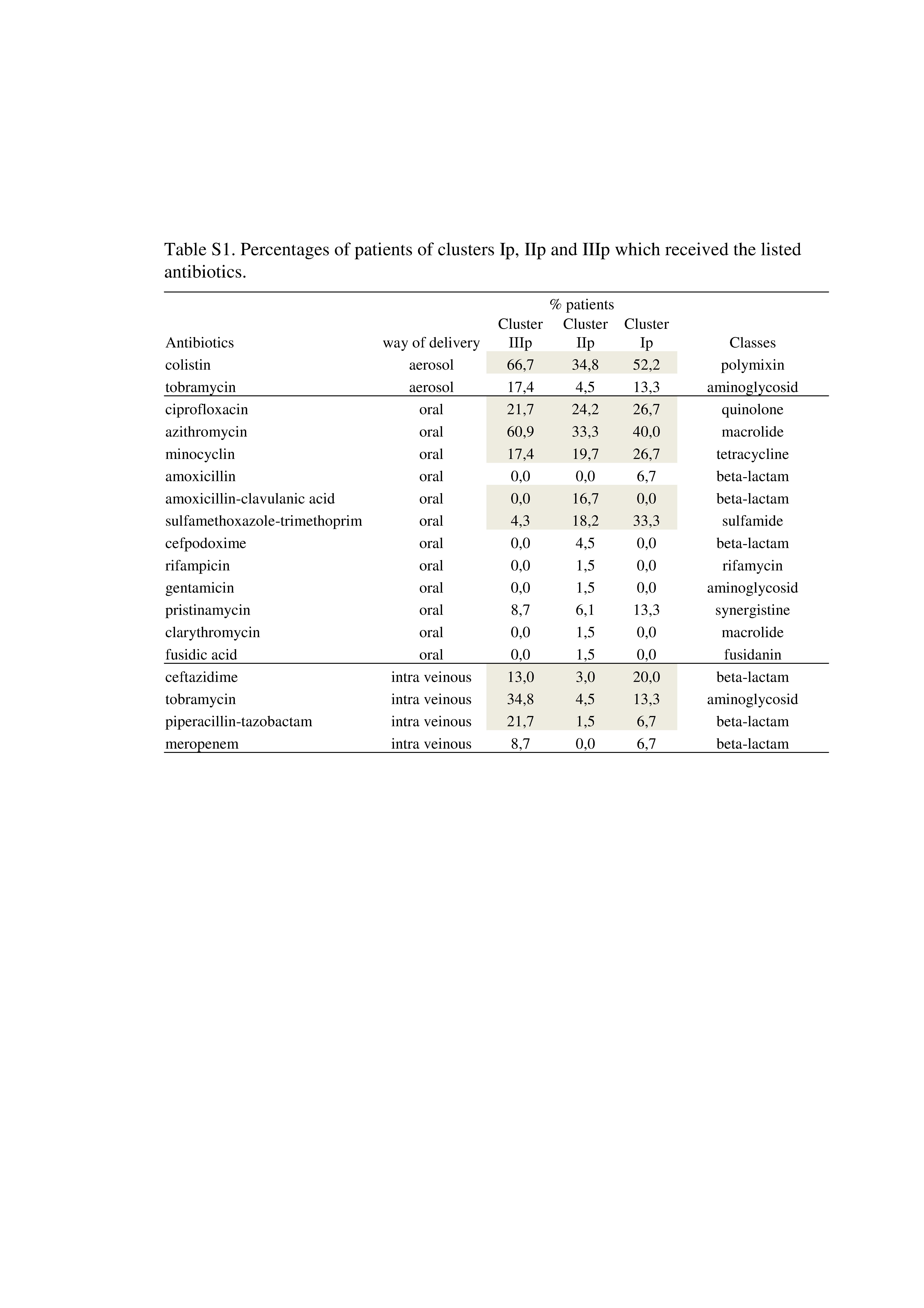

Supplement: S1 Table — (TIF) [file pone.0173022.s001.tif]

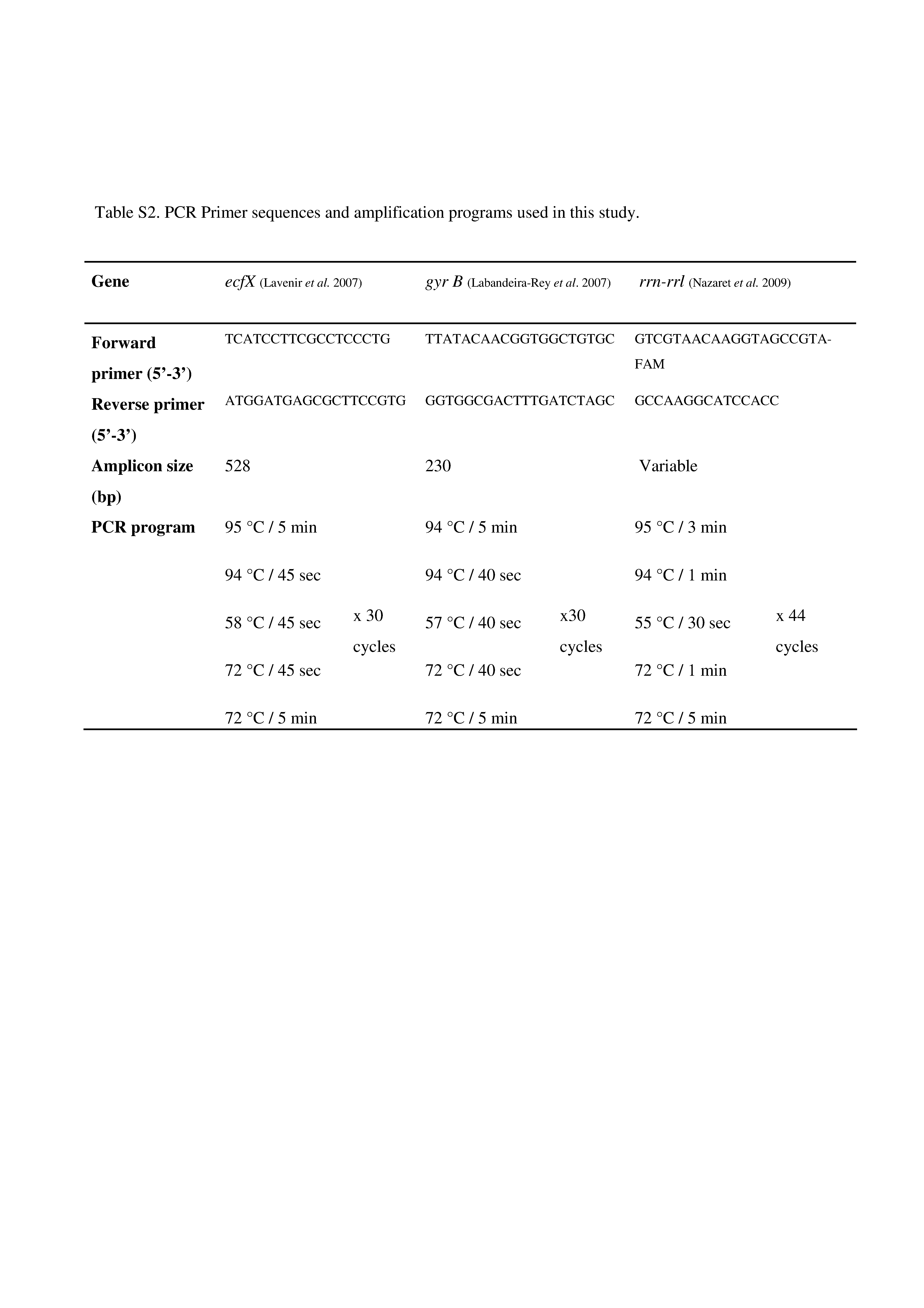

Supplement: S2 Table — (TIF) [file pone.0173022.s002.tif]

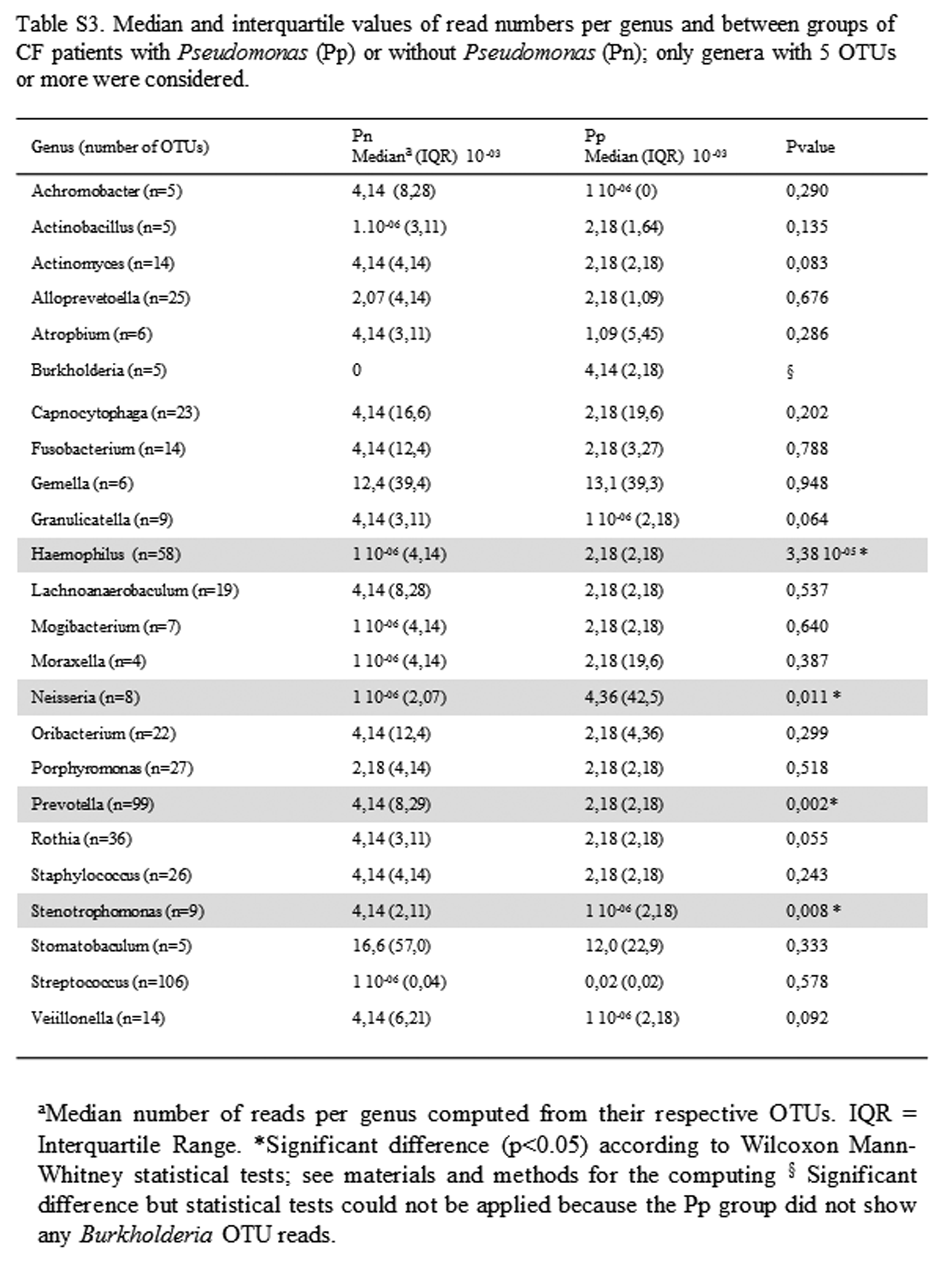

Supplement: S3 Table — (TIF) [file pone.0173022.s003.tif]

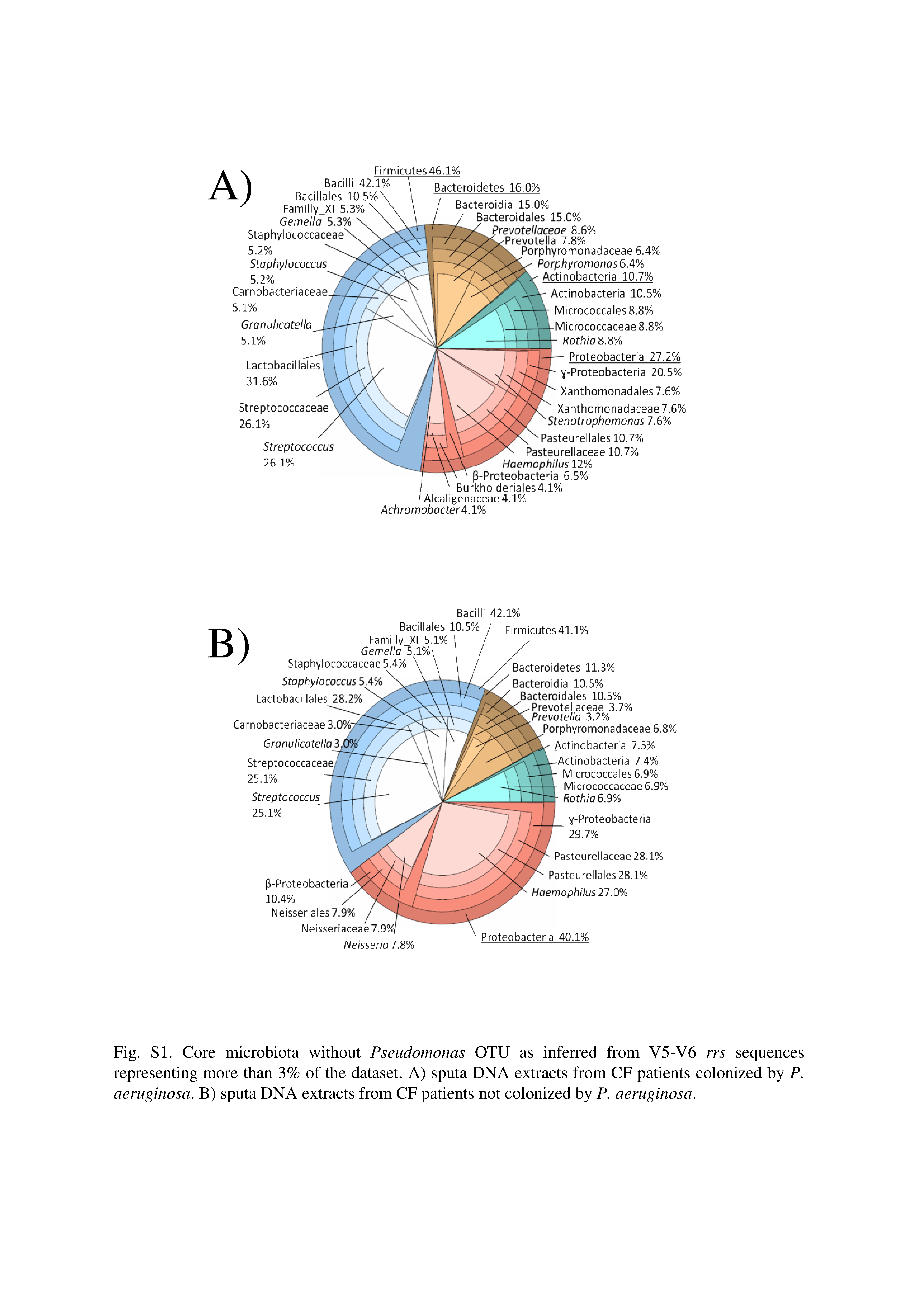

Supplement: S1 Fig — A) sputa DNA extracts from CF patients colonized by P. aeruginosa. B) sputa DNA extracts from CF patients not colonized by P. aeruginosa. (TIF) [file pone.0173022.s004.tif]

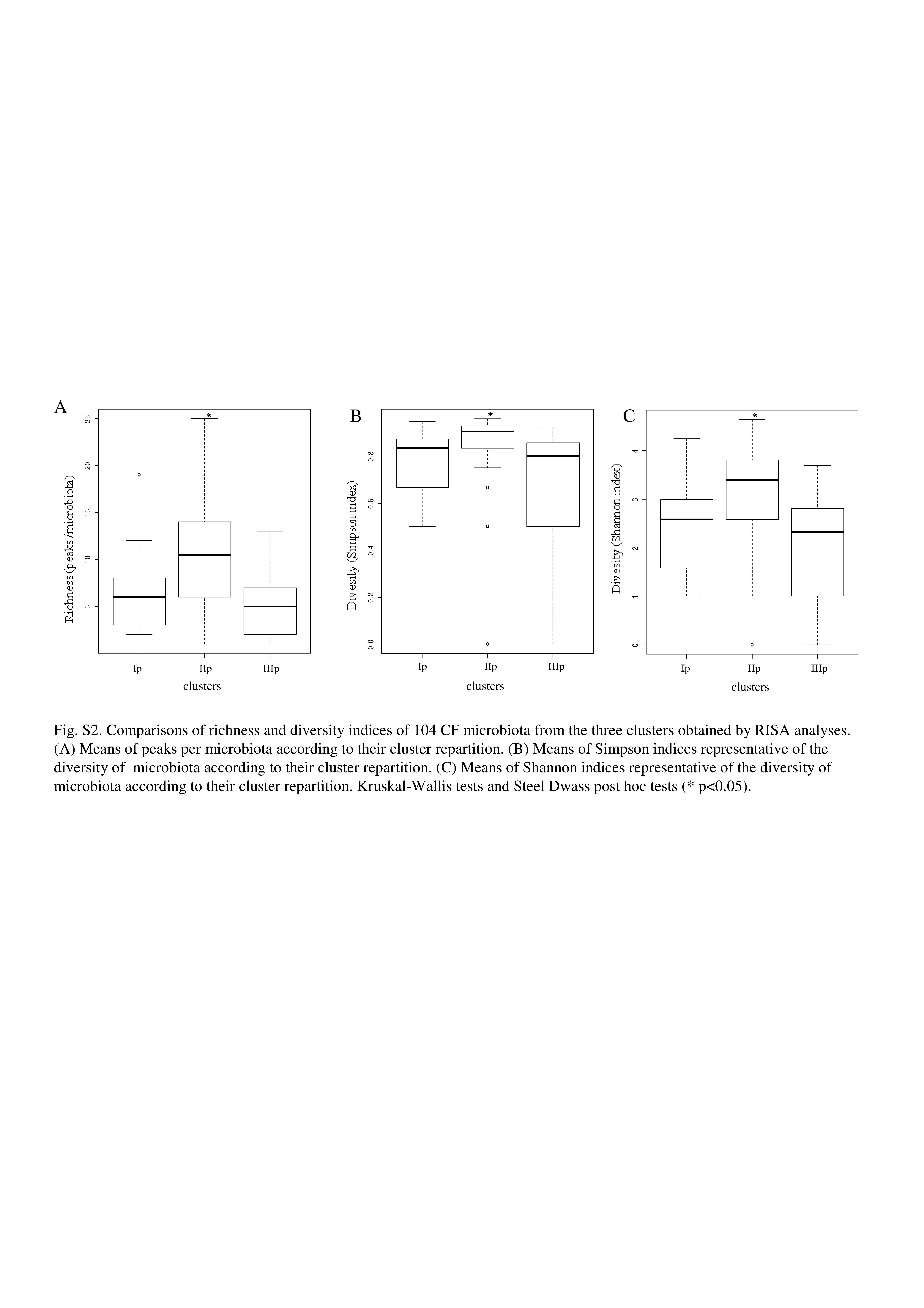

Supplement: S2 Fig — (A) Median of peaks per microbiota according to their cluster repartition. (B) Median of Simpson indices representative of the diversity of microbiota according to their cluster repartition. (C) Median of Shannon index representative of the diversity of microbiota according to their cluster repartition. * significant Kruskall Wallis and Steel Dwass multiple comparison tests (p<0.05*). (TIF) [file pone.0173022.s005.tif]

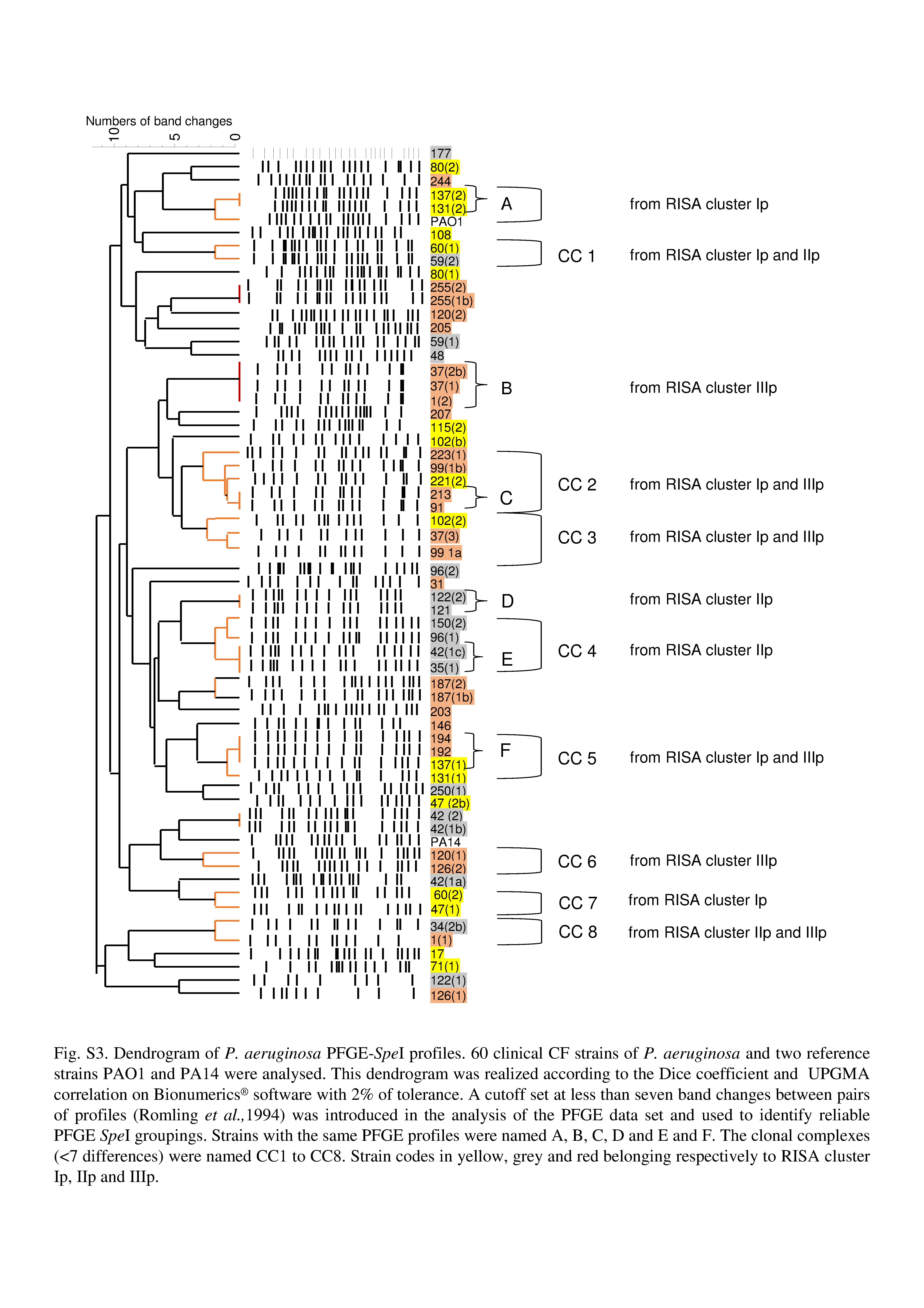

Supplement: S3 Fig — 60 clinical CF strains of P. aeruginosa and two reference strains PAO1 and PA14 were analysed. This dendrogram was realized according to the Dice coefficient and UPGMA correlation on Bionumercis® software with 2% of tolerance. A cutoff set at less than seven band changes between pairs of profiles (Romling et al.,1994) was introduced in the analysis of the PFGE data set and used to identify reliable PFGE SpeI groupings. Strains with the same PFGE profiles were named A, B, C, D and E and F. The clonal complexes (<7 differences) were named CC1 to CC8. Strain codes in yellow, grey and red belonged respectively to RISA cluster Ip, IIp and IIIp. (TIF) [file pone.0173022.s006.tif]
